# Estimating of gases emission from waste sites to generate electrical energy as a case study at Al-Hillah City in Iraq

**DOI:** 10.1038/s41598-023-42335-3

**Published:** 2023-09-14

**Authors:** Ali Chabuk, Udai A. Jahad, Ali Majdi, Hasan S. H. Majdi, Mubeen Isam, Nadhir Al-Ansari, Jan Laue

**Affiliations:** 1 Department of Environment Engineering, College of Engineering, University of Babylon, Babylon, 51001 Iraq; 2 Building and Construction Techniques Engineering, Al-Mustaqbal University College, Babylon, 51001 Iraq; 3 Head of Faculty, Al-Mustaqbal University College, Babylon, 51001 Iraq; 4 Research and Studies Unit, Al-Mustaqbal University College, Babylon, 51001 Iraq; 5 Department of Civil Environmental and Natural Resources Engineering, Lulea University of Technology, 971 87 Lulea, Sweden

**Keywords:** Environmental sciences, Environmental social sciences

## Abstract

Methane (CH_4_) is a greenhouse gas resulting from human activities, especially landfills, and it has many potential environmental issues, such as its major role in global warming. On the other hand, methane can be converted to liquid fuel or electricity using chemical conversion or gas turbine generators. Therefore, reusing such gases could be of great environmental and economic benefit. In this context, this study aims to estimate the emissions of methane gas from the landfills in Al-Hillah City, Iraq, from 2023 to 2070 and the producible electric energy from this amount. The estimating process was carried out using the Land GEM model and compared with traditional models. The obtained results demonstrated that the total estimated landfill methane emissions for 48 years are 875,217 tons, and the average annual methane emission is 18,234 tons based on a yearly waste accumulation rate of 1,046,413 tons and a total waste amount of 50,227,808 tons. The anticipated loads of methane gas can be utilized to generate about 287,442 MW/year of electricity from 2023 to 2070. In conclusion, the results obtained from this study could be evidence of the potential environmental and economic benefits of harvesting and reusing methane gas from landfills.

## Introduction

Global warming is a serious environmental issue caused by greenhouse gas emissions, including methane (CH_4_), carbon dioxide (CO_2_), and nitrous oxide (N_2_O)^1,2^. The evidence of global warming is well proved by the increase in weather temperature, melting of the Arctic Sea ice, weather pattern changes, and insects’ ecology change^3,4^. The estimation of the future emissions of greenhouse gases (GHGs) from developing countries suggests high values that would reach 64% and 76% of total global GHG in 2030 and 2050, respectively^5^. Methane and nitrous oxide have high global warming potentials (GWP) compared to carbon dioxide; the estimated GWP of methane and nitrous oxide are 28–36 and 264–298 times that of carbon dioxide for the next 100 years^6^. Although there are many sources of methane, nitrous oxide, and carbon dioxide, municipal solid waste (MSW) in landfills is one of the major sources of these gases^7–9^. For example, it has been reported that landfills were ranked as the third-largest source of methane in the United States in 2020^10,11^. It should be mentioned here that MSW refers to all materials resulting from different daily activities such as residential, commercial, industrial, institutional, and construction^12,13^. It also should be mentioned that landfills are recognized as one of the most commonly used methods for biodegradable waste disposal in developing countries due to their management simplicity and affordability^14,15^. The anaerobic decompositions of organic waste in the MSW release methane and other GHGs into the atmosphere, and the mounts of the produced GHGs is governed by several factors, including landfill volume, temperature, organic content, moisture, and waste age^16^. Unfortunately, the number of landfills is constantly increasing in developing countries due to the increase in population and waste generation per capita, which in turn results in a significant increase in the production of MSW^17^.

**Figure 1:**
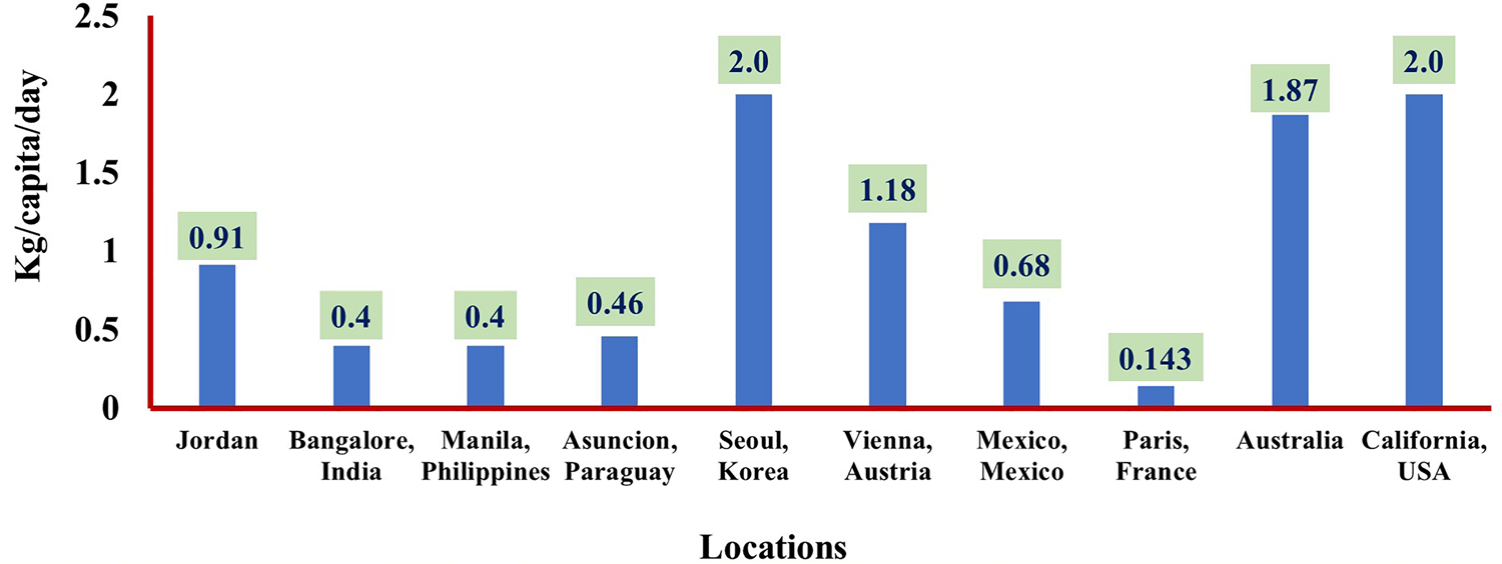
MSW generation rate in different countries^12,18,19^.

Researchers from various regions have studied the MSW generation rates and generally concluded that the MSW generation rate increases with the increase of the income per person, see Fig. 1^12,18,19^.

Like any other developing country, the use of landfills in Iraq is expanding quickly due to the rapid increase in the population, which recently reached 42 million people^12,20^. For example, Alsamawi, et al.^18^ reported that the solid waste generation rate in the capital of Iraq (Baghdad) rates increased from 0.63 kg/capita.day in 2006 to 0.74 kg/capita.day in 2010. Similarly, the solid waste generation in Mosul city, Iraq, in 2010 was 0.647 kg/capita.day, and it would increase within the next few years to reach 1.1 kg/capita.day by 2028^21^. Unfortunately, solid waste management in Iraq is still unstable for many reasons, such as repeated wars and sanctions^22^. Additionally, population development has increased trash production, placing a significant burden on the facilities for handling and disposal^23,24^. Therefore, the expected emissions of GHGs from the landfills in Iraq are expected to be huge within the next few years, which in turn indicates the urgent need to explore efficient GHGs recycling methods.

A significant body of research, therefore, was carried out to minimize the effects of GHGs on the environment through several approaches^25,26^. For example, recycling of methane can be done by converting it into heat or electricity to control its emissions and support the responsible operation of landfills^27,28^. The selection of the best recycling approach depends on the generated amount of GHGs, which can be estimated using several methods. One of the most commonly used methods to estimate the emissions from landfills is the Land GEM developed by the United States Environmental Protection Agency (USEPA)^29–31^.

In this context, this research aims to estimate the total methane emissions from the local landfill in Al-Hillah City, Iraq, for the next 47 years (2023 to 2070) and the potential production of electrical power from the produced methane gas. The estimation process will be carried out using the Land GEM model.

## The study area 

**Figure 2:**
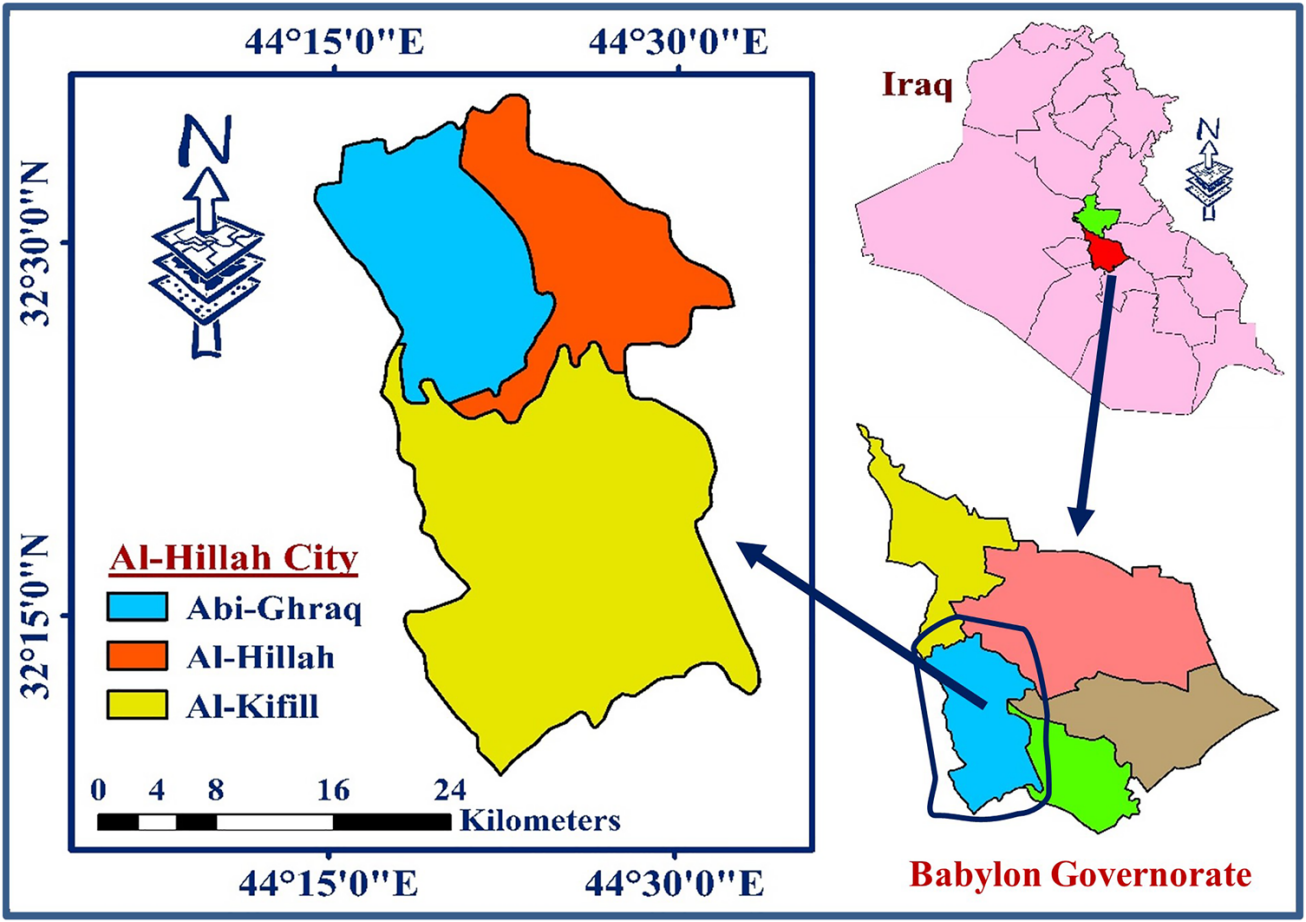
Location of Al-Hillah City.

Al-Hillah city is one of the major cities in Babylon Province (100 km south of the capital city of Iraq^32^), and it is located between latitudes 32°36′1″ and 32°8′45″ North and longitudes 44°14′9″ and 44°33′39″ East (Fig. 2), with a total area of 860 km^2^^12,23^. This city is the home of about 993,000 people, according to governmental reports in 2020^33^, with a population density of 1155 capita/km^2^, which is a relatively high density. Geographically, Al-Hillah city lies between the Tigris and Euphrates Rivers, making it rich in agricultural areas^34^. Typically, the average sunlight in the city is 12 h per day in summer and 6.8 h per day in winter. The city’s climate is dry in summer and cloudy in winter, with average annual relative humidity and rainfall of 45.8% and 102 mm, respectively, and an average annual wind speed of 7.2 km/h. The weather temperatures extremely vary in this city depending on the season and daytime, where it could be less than zero °C in winter and more than 50°C in summer^12,35,36^.

## Classification of solid waste in Al-Hillah City

**Figure 3:**
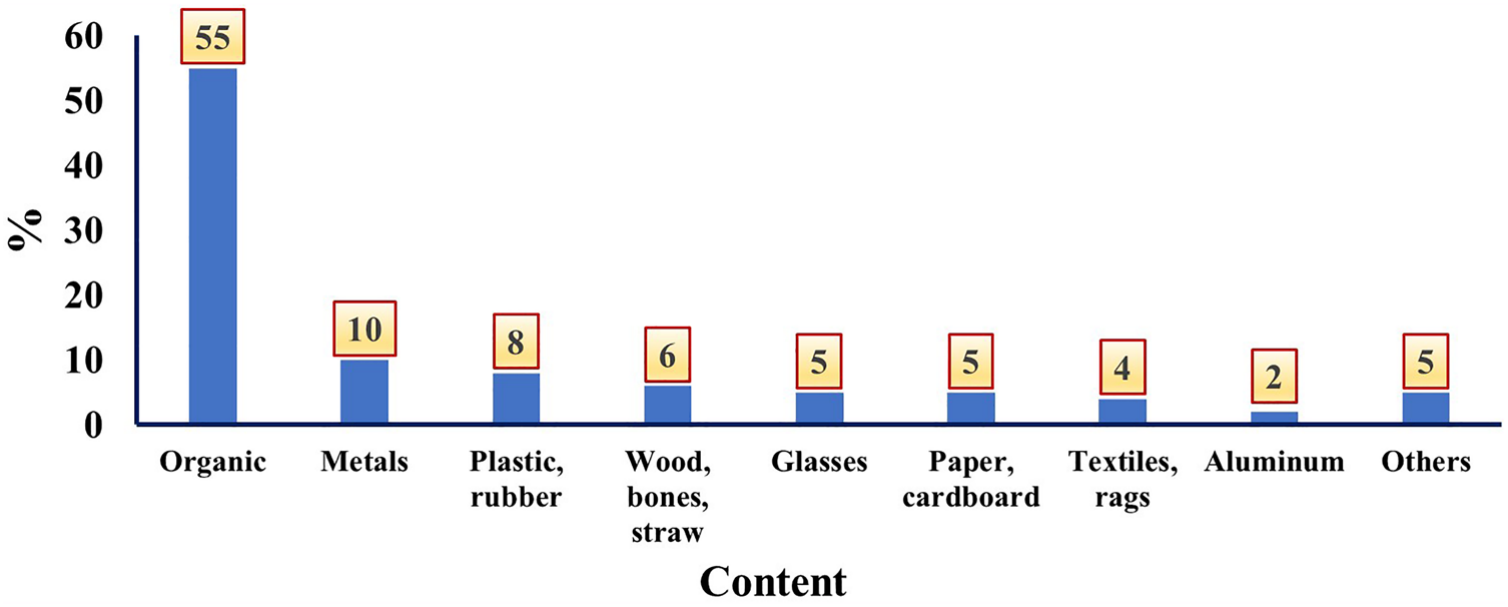
Composition of municipal solid waste in Al-Hillah City^37,38^.

The generated solid wastes in Al-Hillah City are divided into nine categories, namely organic, metals, plastics, wood, glass, paper, textile, aluminum, and others^37,38^. Organic solid wastes are the predominant type of solid waste in Al-Hilla City (55% of the total solid waste in the city), while aluminum represents the lowest percentage (2%). Figure 3 shows the percentages of these nine categories as a function of the total amount of solid waste produced in the city^12,23^.

## Solid waste management in Al-Hillah City 

Currently, solid wastes in Al-Hillah City are manually collected twice a day by the local authorities in the city. Then, the collected wastes are transported to the sorting stations by various waste-collecting vehicles, such as compactors, tractors, large dumpers, and mini-dumpers. The reusable wastes, such as aluminum, cans, and glass, are recovered in the sorting stations. Then, the remaining wastes, mainly organic solid wastes, are either burned or landfilled. Additionally, waste pickers often search wastes for valuable waste components, such as metals and cans, before the collection process to sell these components in the local markets^32,37,38^.

Generally, municipal waste management services at Al-Hillah City cannot manage the whole volume of solid waste; it has been reported that only 44% of the population is served by waste collection services due to the lack of collection sites (there are only four sites in the city)^23,38^. The first site is in the Al-Neel district (17 km to the North of Al-Hillah City), and it is used to collect trash from the Al-Neel and Abi-Ghraq districts. The second site is in the Al-Kifil district (35 km to the South of Al-Hillah City) and has not been effectively utilized. The third site is a transitory site used to gather waste from specific locations within the city before transporting it to the Al-Neel waste site. The fourth site is local and serves only the Al-Kifil district^23,38^.

## Materials and methods

### Calculation method

Initially, the future population of Al-Hillah City in the year 2070 was estimated using Eq. (1), which is necessary to estimate the future solid waste generation in 2070^12,23^.

1 $${\text{P}}_{{{\text{ex}}}} = {\text{ P}}_{{\text{c}}} \left( {{1} + {\text{g}}} \right)^{{\text{y}}}$$

P_ex_ is the estimated population for the selected year. P_c_ is the current population for the starting year. g is the rate of annual growth = 2.99%. y is the years number.

While the generation rate of solid waste (EGRSW) was calculated using Eq. (2)

2 $${\text{EGRSW }} = {\text{ GSW}}_{{({2}0{23})}} \left( {{1 } + {\text{ WGI}}} \right)^{{\text{y}}}$$

EGRSW is the calculated generation rate of solid waste for each year (kg/capita/day). GSW is the current generation rate of solid waste for the year 2023 (0.82 kg per capita per day). WGI is the annual increment rate of waste generation per year (1% according to the literature).

The main equation to calculate the quantity of solid waste (EQSW) generated for every year until the year 2070, based on Eqs. (1) and (2), is^12^:

3 $${\text{EQSW (for specific year) }} = { (}({\text{P}}_{{({\text{current}})}} ({1 } + \, 0.0{299})^{{\text{y}}} ) \, \times {\text{ (GSW}}_{{({\text{current}})}} ({1 } + \, 0.0{1})^{{\text{y}}} {) } \times { (365}/{1}000{))}$$

### Land GEM Model

The US Environmental Protection Agency (USEPA) developed Land GEM software to estimate the rate of gas emissions from municipal dumpsites. The land GEM model is a first-order equation, Eq. (4), which yields the annual emission rate of the targeted gas after calculating two key parameters, namely k (decay rate) and L_0_ (generation potential). The latter parameters can be calculated using either assumptions or actual data. These parameters were calculated in the current study using actual data gathered during a field survey.

The annual methane gas emissions were determined by the following land GEM model^39^:

4 $${\mathrm{Q}}_{\mathrm{CH}4}=\sum_{\mathrm{i}=1}^{\mathrm{n}}= \sum_{\mathrm{j}=0.1}^{1}\mathrm{k }{\mathrm{L}}_{0} ({\mathrm{W}}_{1}/10) {\mathrm{e}}^{-{\mathrm{kt}}_{\mathrm{ij}}}$$

Q_CH4_ is the annually produced quantity of CH_4_ gas (m^3^/year). n is the number of years used in the model for calculating. i is the period increment (1 year). j is the period increment (0.1-year). L_o_ is the potential generation capacity of CH_4_ gas (m^3^/Mg). k is the rate of methane (CH_4_) production (1/year). W_i_ is the accepted waste quantity for interval year (ith) (ton). t_ij_ is the age of waste mass (W_i_) for the part (jth) in the year (ith).

### Governing equations

The capacity to generate methane at a rate of L_0_ (m^3^/Mg) from the landfills in Al-Hillah City depends on the type and contents of solid waste^39^.

Land GEM is built on a first-order decomposition rate equation to estimate emissions from the degradation of landfilled MSW. The model offers a comparatively straightforward method for calculating landfill gas emissions. Methane yield is determined by the model using essential inputs, which are^39^:

(i) The amount of waste dumped in landfills during the studied period.
(ii) Degradable organic content (DOC).
(iii) The form of organic waste.
(iv) Decomposition rate.

5 $${\text{DOC }} = {\text{ S}}_{{1}} \left( {\text{A}} \right) \, + {\text{ S}}_{{2}} \left( {\text{B}} \right) \, + {\text{ S}}_{{3}} \left( {\text{C}} \right) \, + {\text{ S}}_{{4}} \left( {\text{D}} \right) + {\text{ S}}_{{5}} \left( {\text{E}} \right)$$

DOC represents decomposable organic carbon. A represents paper and cardboard trash in MSW. B represents textile waste in the MSW. C represents food waste in the MSW. D represents wood, bones, and straw waste in the MSW. E represents plastic and rubber waste in the MSW. S_1_, S_2_, S_3_, S_4_, S_5_ represent organic carbon that is degraded for each fresh waste type (%).

The decay rate (k) is the half-life of biodegradation of organic waste in landfills, expressed in (1/year). The considerable degree of ambiguity and mistake related to k is acknowledged by the IPCC (2006)^40^. The decay rates in dumps located in arid, chilly areas can be from 1 to 50 years, and sometimes even longer. The following equation is used to estimate the decay rate:

6 $${\text{k }} = { 3}.{2 } \times { 1}0 \, {-}{ 5 }\left( {\text{x}} \right) \, + \, 0.0{1}$$

k represents a decay rate (year^−1^); x represents the yearly mean rainfall for the relevant time for the region where the location of the landfill is.

The value of L_0_ increases with the cellulose waste content. Methane generation potential (L_0_) has a value between 6.2 and 270 m^3^/Mg of waste. The EPA sets L_0_ to a default value of 170 m^3^/Mg waste^39^.

7 $${\text{L}}_{0} = {\text{ MCF }} \times {\text{ DOC }} \times {\text{ DOC}}_{{\text{f}}} \times {\text{ F }} \times { 1}.{334}$$

L_o_ represents the possible capacity of methane production (kg/ton); MCF represents a correction factor of methane, where its default value is equal to 1; DOC represents a degradable organic carbon (kg/ton); DOC_f_ represents an assimilated fraction DOC, where (default of IPCC, 1996 = 0.77; default of IPCC, 2006 = 0.50; F represents the methane gas fraction in a landfill with a default value of 0.5 (Eq. 7); (1.334) represents a conversion factor of methane (molecular weight ratio) to carbon^39^.

By applying the equation of the Land GEM model (Eq. 4), the annual methane gas emissions can be determined for a specific period.

The required input data into the Land GEM model included the values of methane rate (k) = 0.02 (year^−1^), potential methane generation capacity (L_0_) = 100 (m^3^/Mg), methane content = 60 (% by volume), and NMOC concentration = 600 (ppmv as hexane).

## Results and discussion 

The Land-GEM model stands as a critical tool for ensuring the proper management of landfills as it provides a realistic estimation of the generated amount of methane, which provides an accurate picture of the amount of methane and the necessary collection systems. Consequently, this model reduces the risk of explosions and leakage of landfills, and it provides an estimation of the generated heat and power from the harvesting of methane.

The waste generation rates and cumulative waste in place for the waste sites in Al-Hillah City in units of (tons/year) for the period of (2023–2070) were estimated in the Land GEM model. Table 1 shows the estimated weight of solid wastes (tons) for 48 years based on the population in the study area during 2023–2070. Based on Eq. (3), the calculated quantity of EQSW generated for every year until 2070 was calculated using the present generation rate of GSW for 2023 (0.82 kg/capita. day), and WGI equals 1%. Additionally, the predicted population for every year from 2023 until 2070 (P_ex_) with an annual growth rate of 2.99% using Eq. (1).

**Table 1:** Waste generation and cumulative waste in the waste sites in Al-Hillah City (2023–2070).

Year	Population	Total weight (ton/year)	Year	Population	Total weight (ton/year)	Year	Population	Total weight (ton/year)
2023	1,084,531	358,561	2039	1,737,653	673,636	2055	2,784,094	1,265,576
2024	1,116,959	372,974	2040	1,789,609	700,716	2056	2,867,339	1,316,451
2025	1,150,356	387,968	2041	1,843,118	728,884	2057	2,953,072	1,369,371
2026	1,184,751	403,563	2042	1,898,227	758,184	2058	3,041,369	1,424,419
2027	1,220,175	419,786	2043	1,954,984	788,662	2059	3,132,306	1,481,679
2028	1,256,659	436,661	2044	2,013,438	820,366	2060	3,225,962	1,541,241
2029	1,294,233	454,215	2045	2,073,640	853,344	2061	3,322,418	1,603,197
2030	1,332,930	472,474	2046	2,135,642	887,647	2062	3,421,758	1,667,644
2031	1,372,785	491,467	2047	2,199,497	923,330	2063	3,524,069	1,734,682
2032	1,413,831	511,223	2048	2,265,262	960,447	2064	3,629,439	1,804,414
2033	1,456,105	531,774	2049	2,332,994	999,056	2065	3,737,959	1,876,950
2034	1,499,642	553,150	2050	2,402,750	1,039,217	2066	3,849,724	1,952,401
2035	1,544,482	575,387	2051	2,474,593	1,080,992	2067	3,964,830	2,030,886
2036	1,590,662	598,517	2052	2,548,583	1,124,447	2068	4,083,379	2,112,525
2037	1,638,222	622,576	2053	2,624,785	1,169,649	2069	4,205,472	2,197,447
2038	1,687,205	647,603	2054	2,703,267	1,216,668	2070	4,331,216	2,285,782

The methane estimation (ton/year) from landfills in Al-Hillah City for the selected periods (2023–2070) is shown in Table 2. In the Land GEM model, the annual generated rate of total methane was 1.32 × 10^–3^ tons per 1 tan of solid waste from the landfills in Al-Hillah City.

**Table 2:** Total methane emissions from landfills in Al-Hillah City for the studied period.

Year	Methane (ton/year)	Year	Methane (ton/year)	Year	Methane (ton/year)	Year	Methane (ton/year)
2023	456	2035	6820	2047	15,700	2059	29,000
2024	921	2036	7450	2048	16,700	2060	30,400
2025	1400	2037	8090	2049	17,600	2061	31,900
2026	1880	2038	8760	2050	18,600	2062	33,300
2027	2380	2039	9440	2051	19,600	2063	34,900
2028	2890	2040	10,100	2052	20,600	2064	36,500
2029	3410	2041	10,900	2053	21,700	2065	38,200
2030	3940	2042	11,600	2054	22,800	2066	39,900
2031	4490	2043	12,400	2055	24,000	2067	41,700
2032	5050	2044	13,200	2056	25,200	2068	43,500
2033	5620	2045	14,000	2057	26,400	2069	45,500
2034	6220	2046	14,900	2058	27,700	2070	47,500

According to Al-Rawi (2013)^41^, 6280 tons of methane gas can generate 11,304 kWh, which was used in the equation below to estimate the producible electricity power (GEP) (kW/year) from methane gas generation (MGG) in 2023:

$${\text{GEP}}_{{{2}0{23}}} \left( {{\text{kW}}/{\text{year}}} \right) = {\text{ MGG}}_{{{2}0{23}}} \left( {{455}.{8}} \right) \, \left( {{\text{ton}}/{\text{year}}} \right) \, \times { 113}0{4 }\left( {{\text{kWh}}} \right) \, \times { 24 } \times { 365 } = { 7187}0{\text{54 kW}}/{\text{year}} = { 7187}.0{\text{54 MW}}/{\text{year}}$$

Table 3 shows the generated methane gas from the landfills in Al-Hillah City in the next 47 years and the producible electrical power in MW/year. The estimated amount of accumulated waste in the next 48 years is about 50,227,808 tons, which equals an average of 1,046,413 tons per year. This amount of waste produces 875,217 tons of methane gas, which equals an average of 18,234 tons of methane per year. Regarding energy production, about 13,797,190 MW/year of electricity could be produced from the methane gas. According to a statement from the Department of Electricity in Babylon, the current need for emergency electric power is projected to reach 100 MW in 2022, which means that recycling up to 65% of the emitted methane gas from landfills will supply 20% of the electric power used in the city. Thus, the idea of re-utilizing the methane emissions in Al-Hillah City is overwhelmingly beneficial in both environmental and economic aspects. Not only would this process be immensely economical, but it would also help the environment in numerous ways by reducing the amount of pollutants in the air. It is noteworthy to highlight that the obtained results about utilizing the GHGs from landfills to produce energy are agreed with the final conclusions of a wide body of literature, such as the studies of Yi et al.^42^, Ghosh et al.^43^, Yaman^44^, Tan et al.^45^, Dace et al.^46^ and Nabavi-Pelesaraei et al.^47^.

**Table 3:** GEP (MW/year) for 2023 – 2070 using the emitted methane from the landfills in Al-Hillah City.

Year	Methane (ton/year)	GEP (MW/year)	Year	Methane (ton/year)	GEP (MW/year)	Year	Methane (ton/year)	GEP (MW/year)
2023	456	7190	2039	9440	149,000	2055	24,000	378,000
2024	921	14,500	2040	10,100	160,000	2056	25,200	397,000
2025	1400	22,000	2041	10,900	171,000	2057	26,400	416,000
2026	1880	29,700	2042	11,600	183,000	2058	27,700	437,000
2027	2380	37,500	2043	12,400	195,000	2059	29,000	458,000
2028	2890	45,500	2044	13,200	208,000	2060	30,400	480,000
2029	3410	53,700	2045	14,000	221,000	2061	31,900	502,000
2030	3940	62,100	2046	14,900	234,000	2062	33,300	526,000
2031	4490	70,700	2047	15,700	248,000	2063	34,900	550,000
2032	5050	79,600	2048	16,700	263,000	2064	36,500	575,000
2033	5620	88,700	2049	17,600	277,000	2065	38,200	602,000
2034	6220	98,000	2050	18,600	293,000	2066	39,900	629,000
2035	6820	108,000	2051	19,600	309,000	2067	41,700	657,000
2036	7450	117,000	2052	20,600	325,000	2068	43,500	686,000
2037	8090	128,000	2053	21,700	342,000	2069	45,500	717,000
2038	8760	138,000	2054	22,800	360,000	2070	47,500	749,000

In conclusion, it could be said, according to the results of this study, this simple solution could pave the way for more efficient and sustainable energy production in the future.

## Conclusions 

Solid waste accumulation and the shortage of electrical power generation are two critical issues Iraq faces. In an effort to address them, this study was conducted to find a sustainable solution to these issues. The study has indicated the importance of properly managing MSW, which is essential for environmental protection, while the development of alternative energy sources can help fill the increasing gap between electricity production and consumption.

According to the obtained results from the Land GEM model, the amount of accumulated waste over 47 years is 50,227,808 tons, with an annual average of 1,046,413 tons. The generation of this amount of waste has also led to a massive amount of methane emission (about 875,217 tons in total and an annual average of 18,234 tons). The good news is this amount of gas can produce 13,797,190 MW/year of electricity. That’s enough energy to power a large number of emergency services, hospitals, and businesses. In general speech, it can be said that harvesting 65% of the emitted methane gas from the landfills in Al-Hillah City can cover up to 20% of the electricity used for emergency power lines in the city. Not only would this drastically reduce energy expenses, but it could also help purify the air by reducing the amount of pollutants.

To further assess the impacts of the model’s estimates on the environment, data regarding local policies and regulations should be incorporated. This additional information may support the implementation of strategies to successfully improve landfill management and reduce future emissions in Al-Hillah City.

## Data Availability

The data sets can be provided by the corresponding author upon reasonable request.
